# Early onset of capsular contracture after breast augmentation with implant: report of two cases & review of literature

**DOI:** 10.1080/23320885.2022.2077207

**Published:** 2022-05-23

**Authors:** Subhi M. K. Zino Alarki, Hatan Mortada, Asma I. Abdullah, Hisham Alkhalidi, Musab Alrehaili

**Affiliations:** a Department of Surgery, Division of Plastic Surgery, King Saud University Medical City, King Saud University, Riyadh, Saudi Arabia; b Department of Plastic Surgery and Burn Unit, King Saud Medical City, Riyadh, Saudi Arabia; c Department of Pathology, College of Medicine, King Saud University, Riyadh, Saudi Arabia

**Keywords:** Capsular contracture, breast reconstruction, early-onset, capsulectomy, complications

## Abstract

Capsular contracture (CC) is a local complication caused by an inflammatory reaction that leads to fibrosis. CC commonly occurs after one year of surgery. Hence, there has been no previously reported early onset of CC. Therefore, we report two unusual cases of early onset of post-breast reconstruction capsular contracture.

## Introduction

Breast reconstruction following mastectomy can be performed any time after mastectomy. It can be implemented as a one-step procedure by placing a permanent implant or as a two-step procedure with the initial tissue expander placement followed by the placement of a permanent implant [1]. As with any surgery, implant-based breast reconstruction has been associated with several risks and complications. The most common complication is capsular contracture [2]. The introduction of nonbiologic materials into the body induces the formation of a capsule. However, in the breast, this may be particularly critical. Capsular contracture is the capsule's thickening and hardening, resulting in a local complication caused by an inflammatory reaction that leads to fibrosis and manifests as firm, deformed and painful breasts that eventually require reoperation [3]. The Baker classification system is used to classify capsular contracture. It is a subjective classification system based on the clinical findings. The classification is divided into four classes: Class I and II are not clinically significant. Class I describes a breast with almost a natural look and feel. Class II describes a breast with minimal contracture in that a surgeon tells the surgery has been performed but with no or very minimal firmness. However, Class III-IV are the ones that carry clinical significance. Class III describes moderate contracture with some firmness felt by the patient, whereas class IV describes severe contracture, which is evident from observation and symptomatic in the patient [4]. The rate of capsular contracture varies according to different variables and from a study to another. Those variables can be the length of follow-up, the type of implant, and the surgical technique used ^[^5]. Over reviewing the literature, we found that the rate of capsular contracture ranges between as low as 2.8–20.4% (Table 1) [6–12]. Despite this wide incidence rate, it is still considered the most common complication following implant-based breast surgeries [12]. Despite the increase in the incidence of capsular contracture, there were no previous case reports on the early onset of capsular contracture secondary to breast augmentation with breast implants. Therefore, this paper is the first that aims to review the current literature available regarding the time frame of the onset of capsular contracture and to report two cases in the form of a case study in terms of how early they developed capsular contracture.

**Table 1.** Demonstrate the reported incidence rates for capsular contracture in the literature.

Study name	Type of implant	Average follow up time (yr)	Incidence of capsular contracture (%)
Spear et al. 2014 [6]	Natrelle, round silicone	6	18.9
Blount et al. 2013 [7]	Various	14.9 mo	2.8
Stutman et al. 2012 [8]	Various	2.4	7.6
Codner et al. 2011 [9]	Various	6	8.2
Y. El-Sheikh et al. 2008 [10]	Cohesive gel silicone	Not mentioned	13.6
Sevin et al. 2006 [11]	Textured silicone gel round implants	8	8
Gutowski et al. 1997 [12]	Saline filled implants	6	20.4

## Case presentation

### Case study – 1

**Figure 1.**
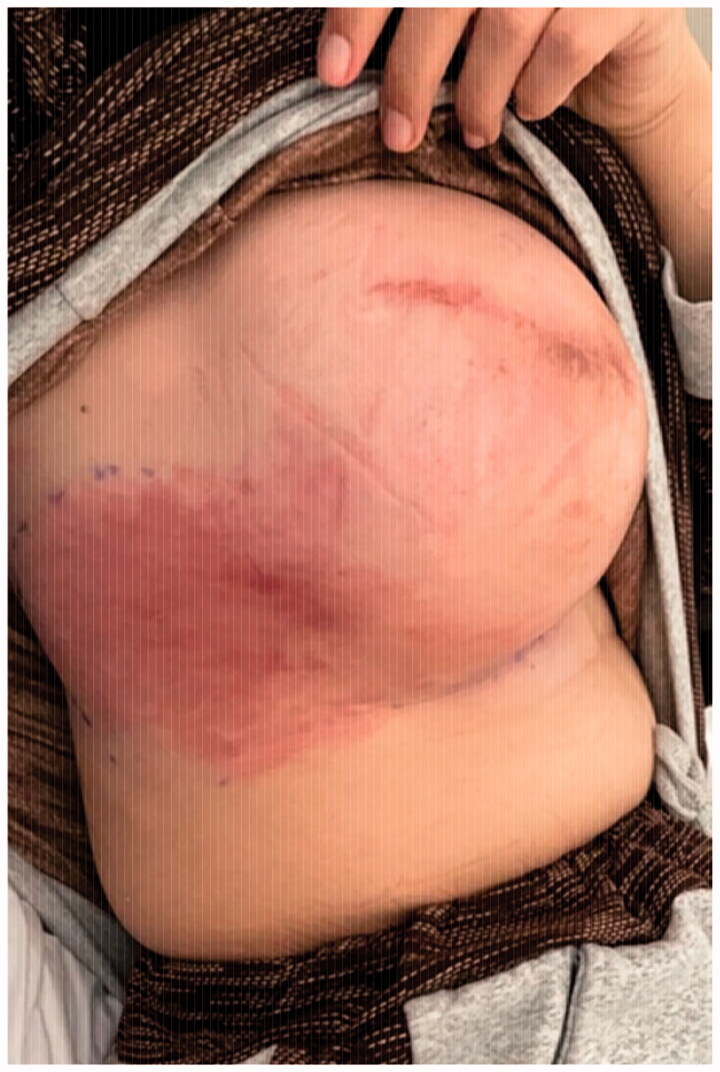
Overlying erythema on the reconstructed breast.

A 43-year-old female patient with a history of right breast cancer underwent right mastectomy and breast reconstruction with a Becker implant for eight years. No history of radiotherapy. She had implant exchange surgeries twice at different intervals previously. The last was on the 11 October 2020, as she was not satisfied with the right breast size with no other indications such as trauma or implant rupture. Therefore, the implant was exchanged with a 550-cc smooth Motiva® silicone implant and discharged afterward without any complications. A capsulotomy was done. Afterward, she presented to our clinic after nine weeks complaining of pain and redness over the right breast with a heaviness extending to the back and shoulder. On examination, the surgical site looked clean and healed—no noticeable discharge, signs suggesting undergoing infection or implant rupture (Figure 1).

**Figure 2.**
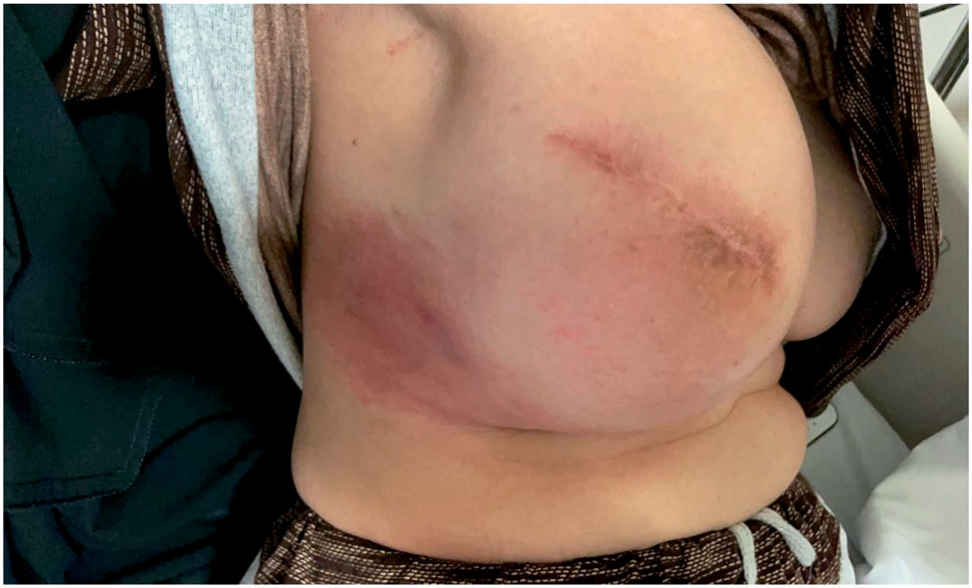
Slight improvement in clinical signs after intravenous antibiotics administration during her admission.

**Figure 3.**
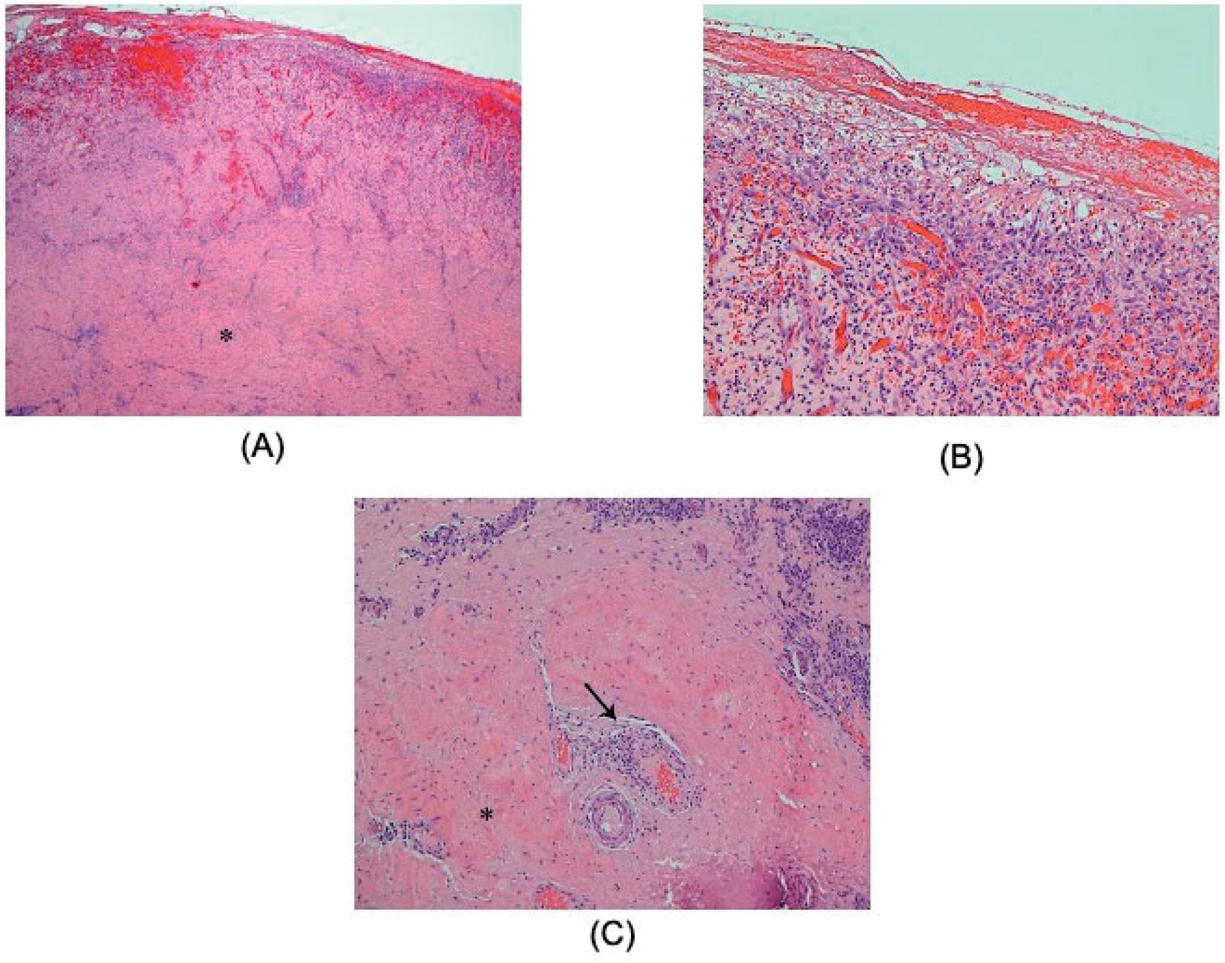
(A) The inner lining of the capsule is eroded and essentially replaced by inflamed granulation tissue, indicating an active organizing inflammatory process. The outer part of the capsule (asterisk) has a dense fibrous stroma (H&E ×20). (B) A high-power view of the inner capsule lining shows inflamed granulation tissue with neovascularization, fibroblast proliferation, acute and chronic inflammatory cells (including macrophages) and fibrin deposits on the surface (H&E, ×100). (C) The outer part of the capsule is fibrotic with dense, thick collagen deposition (asterisk). It also shows scattered foci of chronic perivascular inflammation (arrow), with congestion and vascular dilatation (H&E, ×100).

**Figure 4.**
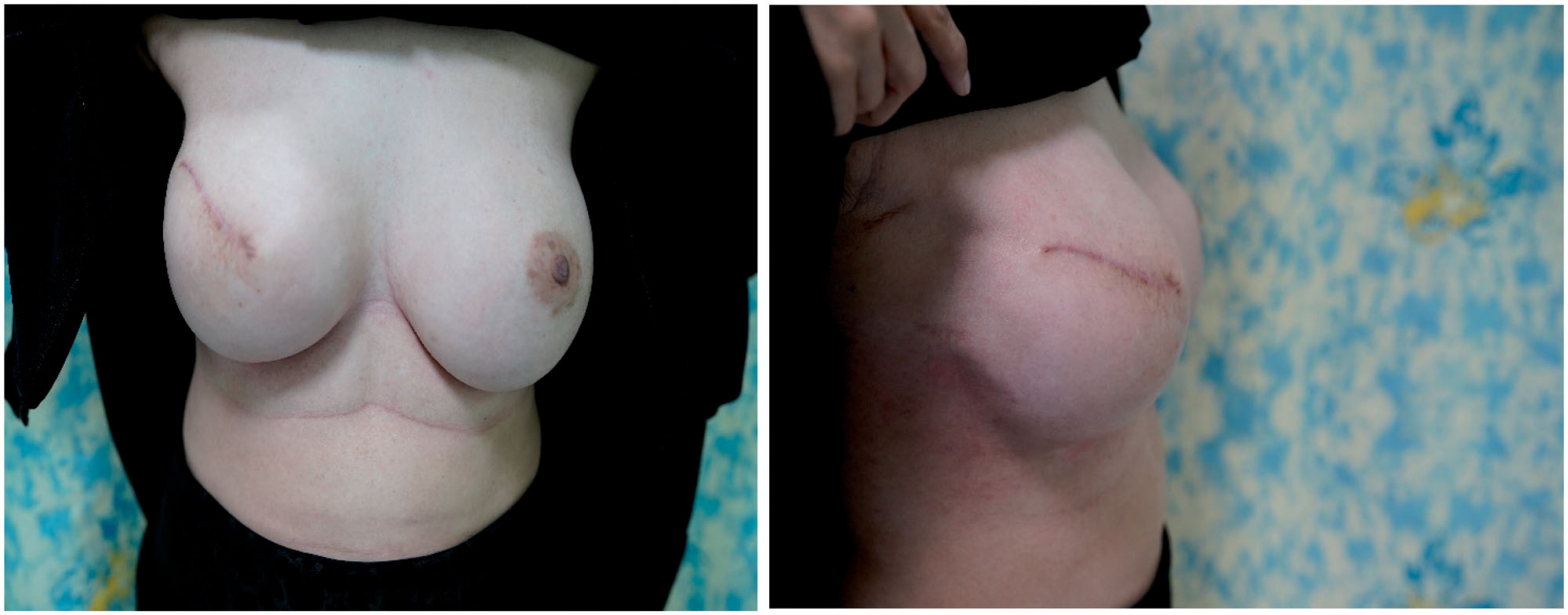
(a,b) Dramatic improvement postoperatively on her follow up visit.

Consequently, right breast ultrasonography was done. It yielded the impression that there were no edematous changes, signs of inflammation, and no drainable fluid collections. The patient was admitted for further evaluation and to start intravenous antibiotics and MRI arrangement. After starting her empirically on clindamycin, the patient's clinical status slightly improved. However, erythema was still evident over the right breast (Figure 2). The MRI reported the presence of a double-lumen retro mammary implant, which is suggestive of capsular contracture. After that, the patient underwent capsulectomy and implant exchange. Intraoperatively, there were no signs of implant rupture, the contracture was confirmed, and capsulectomy was done with the exchange of the implant using the same plane (submuscular pocket with SurgiMend® acellular dermal matrix). The capsulectomy specimen was sent to histopathology. The result showed features of a fibrocystic wall with granulation tissue formation and no malignancy (Figure 3). Symptoms improved significantly, and she was satisfied with the result. The erythema disappeared (Figure 4(a,b)).

### Case study – 2

**Figure 5.**
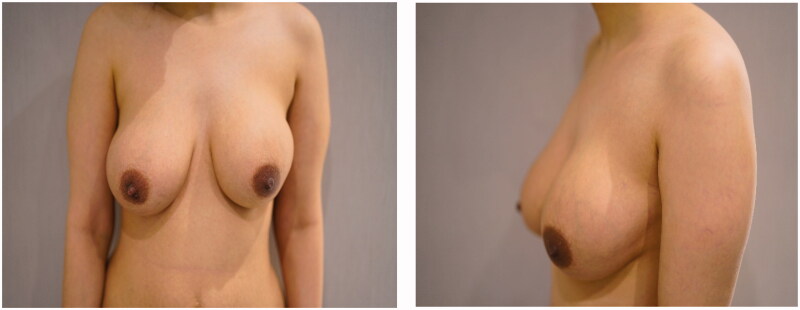
(a,b) Obvious distortion in the shape of the left breast.

**Figure 6.**
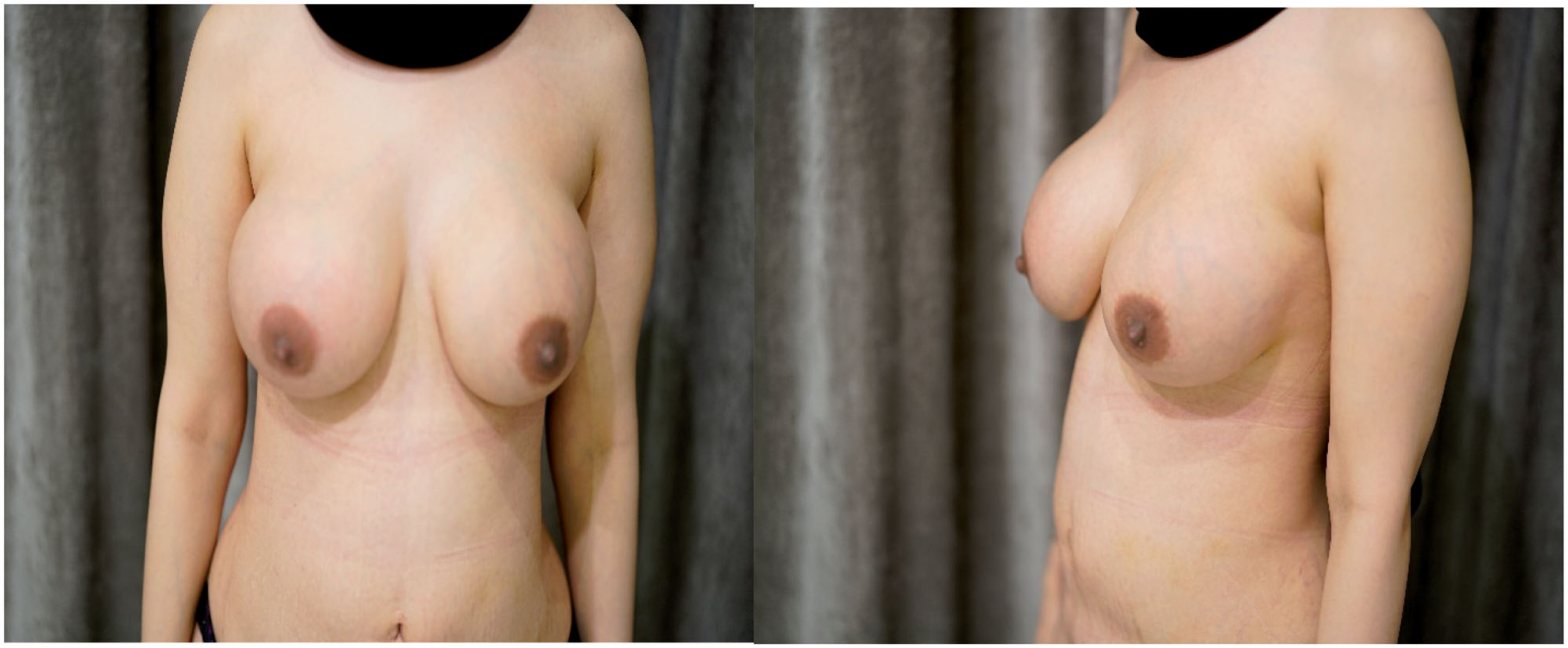
(a,b) Dramatic improvement for the left breast signs with maintain a good symmetry.

A 27-year-old female patient, generally healthy. After eight weeks of undergoing bilateral breast augmentation using micro-textured POLYTECH ® silicone implants due to small bilateral breasts. She presented to the clinic with noticeable changes in her left breast and increased hardness (Figure 5(a,b)). She was not complaining of pain. The laboratory and clinical findings were not significant as there was no increase in inflammatory markers or cutaneous signs of infection. Breast ultrasonography and MRI were unremarkable. Subsequently, implant exchange was done. Intraoperatively, there was no evidence of hematoma or infection. However, a severely contracted capsule was observed. Post capsulectomy and breast implant exchange using the same subglandular plane. The capsular contracture was confirmed via histopathology examination. The patient showed dramatic improvement and resolution of the main complaint (Figure 6(a,b)).

## Consent

Written informed consent was obtained from the patients for publication of this case report and accompanying images.

## Discussion

Capsular contracture is a well-established postoperative adverse effect for cosmetic and reconstructive purposes in breast implant surgeries [13,14]. After breast implant surgery, capsule formation is widespread, and all breast implants develop fibrous and scar tissue around it. In some cases, disproportionate fibrosis and scar tissue shrinkage (capsular contracture) causing a specific defect and breast alteration after augmentation or reconstruction. Radiation therapy is associated with a higher rate of capsular contracture. This would be predicted. The effect of RT on normal tissue causing extensive fibrosis among breast reconstruction patients ranged from 17–86% when postoperative RT was obtained [15–21]. As in the author's case, the patient in case one did not receive a previous radiotherapy exposure. In the literature, capsular contracture most commonly occurs after the first and second years post-surgery [22]. Hence, we reported two unique cases that developed capsular contractures nine weeks and eight weeks after their most recent breast surgery. One patient had undergone alloplastic breast reconstruction eight years prior, and the other patient underwent cosmetic augmentation. There is no description of what defines ‘early’ presented regarding the onset of capsular contracture in the literature. The authors believe that capsular contracture occurs within the first six months after surgery and is considered an early onset. As demonstrated in Table 1, the average years of follow-up for the occurrence of capsular contracture ranged from 14.9 months to 8 years. Patients who underwent breast implant surgery are widely fulfilled with the outcome and aesthetic result, whether for augmenting or reconstructive reasons [13,14]. Even yet, it is the surgeon's responsibility to appropriately counsel and advise the patient about the possibility of unwanted complications that may result in unfavorable outcomes. Seroma, hematoma, scarring, infection, breast asymmetry, necrosis, and, most critically, capsular contracture are the most prevalent consequences [22–28]. The breast implant is too large to be successfully phagocytized by the body, as it would be with a much smaller embedded foreign body, resulting in the development of capsular contracture. On the other hand, silicone is too inert to create a toxic reaction because it lacks an active binding site [29]. As a substitute, the foreign implant is encased in a fibrous capsule consisting of myofibrils and collagen. Usually, it does not exceed the thickness of 1 − 1.5 mm [17,18]. The creation of a capsule is considered a physiological component of the healing process, and few studies have suggested that it may even support keeping the implant in place [18,21]. Capsular contracture is a condition that occurs when the capsule thickens and the dimensions of the implant change. The surrounded capsules alter the breasts from their aesthetic appearance, making them feel rigid, even hard, and uncomfortable [18,29]. As this was the primary patient's complaint in our two reported cases. The capsule pulls the centripetal force around the implant, transforming the normally appeared shape into a sphere with the least surface area to volume ratio [22]. This causes spherical breasts, a condition known as capsular contracture, which is identical to the effects of pushup bra [22]. Different circumstances, such as the surgical indication of the pocket creation, raise one's risk of developing capsular contracture, and the incidence in such a group will generally be higher. The risk is higher if a sub-glandular pocket is indicated for the patient [27]. Post-breast implant surgery can develop capsular contracture, which leaves a patient with painful, stiff, spherical breasts. Thus, the diagnosis of capsular contracture should be considered even if it was early onset after surgery.

The lack of diagnostic criteria for capsular contracture may limit the ability to know the true incidence of capsular contracture. Hence, capsular contracture is diagnosed clinically, that is often missed on imaging [30]. On MRI, capsular contracture may not always show findings; however, an increased anterior-posterior diameter with an increased number of radial folds and thickening of the echogenic fibrous capsule can be detected [31].

Regarding the thickness of the implant capsule, a study conducted by Siggelkow et al. found that the capsule thickness ranged from 0.83 to 1.21 mm [31]. Compared to our study, the capsule thickness of the first case was 6–9 mm. This could explain how capsule thickness can differ significantly according to Baker's classification [32]. As a result, capsular contracture is supposed to be addressed even if it presents within weeks after the surgery with symptomatic painful breasts.

## Conclusion

In conclusion, we report two rare cases of early-onset capsular contracture. Although the difference in implant type was used in both reported cases, early capsular contracture was observed. Immediate diagnosis and treatment of capsular contracture are needed. It can be a highly distressing complication of breast implant surgery; therefore, the diagnosis of capsular contracture should be considered even if it was early-onset after surgery as it can present with spherical, hard, and painful breasts.

## Ethical approval

All procedures performed in studies involving human participants were in accordance with the ethical standards of the institutional and/or national research committee and with the 1964 Helsinki declaration and its later amendments or comparable ethical standards. The authors have granted consent from the patients to use the images.
